# Intuitive Eating Intervention in Physically Active Adults: Effects on Anthropometry, Athletic Performance, Eating Attitudes, and Body Image

**DOI:** 10.3390/nu17172824

**Published:** 2025-08-29

**Authors:** Meltem Pırıl Şenol, Ece Öneş, Murat Baş, Gözde Arıtıcı Çolak

**Affiliations:** 1 Institute of Health Sciences, Acibadem Mehmet Ali Aydinlar University, Istanbul 34752, Türkiye; merhaba@pirilla.com; 2 Pirilla Nutrition Consultancy, Istanbul 34365, Türkiye; 3 Department of Nutrition and Dietetics, Faculty of Health Sciences, Acibadem Mehmet Ali Aydinlar University, Istanbul 34752, Türkiye; murat.bas@acibadem.edu.tr (M.B.); gozde.aritici@acibadem.edu.tr (G.A.Ç.)

**Keywords:** athletic performance, body composition, body image, eating attitudes, exercise, intuitive eating

## Abstract

**Background/Objectives**: There is growing interest in non-diet approaches to support health, well-being, and performance in different populations. The aim of this study was to evaluate the effects of a 12-week intuitive eating (IE) intervention on participants’ body composition, body image, eating behaviors, and athletic performance. **Methods**: The study included both an intervention group and a control group. It was conducted between September and December 2021. Participants were recruited from a sports center in Istanbul, where they had applied for nutrition and exercise counseling. Inclusion criteria included being 18–65 years old, not having engaged in regular physical activity in the past month, having no chronic disease, and not using any regular medications. Participants were not randomly assigned to groups; allocation was based on availability and willingness to attend the intervention sessions. The study involved 57 participants who were healthy adults between 18 and 65 years old and followed a structured exercise program. At the beginning of the study, a demographic questionnaire was administered. The anthropometric measurements were taken at the beginning and at the end of the intervention. In addition, validated performance and psychometric assessment tools were used, including the Cooper test for cardiovascular endurance, the Davies test for upper-body agility, and the 1-RM bench press for muscular strength, alongside standardized self-report questionnaires for eating attitudes (EAT-26), IE (IES-2), and body image (BCS). **Results**: The intervention group did not show any statistically significant changes in body composition (*p* > 0.05). The post-intervention means of the intervention and control groups were not statistically different (*p* > 0.05). The intervention group showed significant improvements in cardiovascular endurance, agility, and strength performance scores compared to the control group after the intervention (*p* < 0.05). The intervention group showed significant improvements in body image scores (*p* < 0.05) and eating attitude scores (*p* < 0.05). The post-intervention eating attitude and body image scores of the intervention group were significantly different from those of the control group (*p* < 0.05). The results of the correlation analysis showed a significant positive correlation between intuitive eating and body image (r = 0.455; *p* < 0.05) and a significant negative correlation between IE and disordered eating attitudes (r = −0.449; *p* < 0.05). **Conclusions**: These findings suggest that longer-term interventions may be beneficial and warrant further investigation. IE may serve as a promising strategy to enhance psychological well-being and performance outcomes without focusing on weight control.

## 1. Introduction

Traditional weight-centric methods in health and fitness have encountered rising criticism for their potential role in fostering disordered eating behaviors, body dissatisfaction, and unsustainable dietary practices [1]. The non-diet weight-neutral framework of IE guides people to eat based on their internal physiological signals instead of following external rules or restrictions [2]. Emerging evidence also suggests that IE may positively influence body composition and metabolic health, independent of total energy intake. IE encourages individuals to rebuild a healthy relationship with food and their bodies through self-awareness, emotional regulation, and body respect [1].

Recent meta-analytic evidence supports the view that IE is linked with a variety of beneficial psychological outcomes. According to the study by Linardon et al. [3], individuals who eat in accordance with their internal hunger and satiety signals are less likely to experience weight and shape concerns, to internalize societal appearance ideals, or to engage in maladaptive eating behaviors such as binge eating, emotional eating, or purging. This suggests that IE may act as a protective factor against disordered eating and body image disturbances. Furthermore, IE was found to be significantly associated with adaptive constructs such as positive body image, self-esteem, self-compassion, and life satisfaction and negatively associated with depressive and anxiety symptoms. These associations indicate that honoring biological cues and approaching eating with self-permission and flexibility contribute to improved mental health and emotional well-being [3].

Moreover, IE has been empirically linked to greater interoceptive sensitivity—that is, the ability to accurately perceive internal bodily cues such as hunger and fullness—which in turn contributes to healthier body weight regulation. A mediation analysis by Herbert et al. [4] demonstrated that interoceptive sensitivity significantly mediates the relationship between IE and body mass index (BMI), highlighting the importance of the brain–body connection in intuitive eating-related outcomes.

The IE model consists of four core components: (1) unconditional permission to eat, (2) eating for physical rather than emotional reasons, (3) reliance on hunger and satiety cues, and (4) body–food choice congruence. Research studies have established that higher intuitive eating scores correlate with lower disordered eating and better psychological well-being and improved body satisfaction [5,6].

Physically active individuals have unique nutritional and physiological demands that distinguish them from general or clinical populations. Their elevated energy expenditure requires sufficient caloric intake and balanced macronutrient distribution to support recovery, muscle maintenance, and performance adaptation. However, pressure to achieve specific body compositions—especially in competitive or appearance-focused environments—may increase vulnerability to disordered eating behaviors, including restrictive dieting, compulsive exercise, and body dissatisfaction. Studies have shown that athletes and recreational exercisers may present with subclinical eating disturbances despite appearing healthy or fit, highlighting the need for early interventions that address both physical and psychological well-being [7]. Within this context, IE may serve as a protective approach by promoting internal regulation, rejecting diet culture, and encouraging sustainable health behaviors in active populations. However, research on the physiological and performance-related effects of IE—especially among physically active, adult populations—remains limited.

Most existing studies have focused on women, adolescents, or clinical samples, often assessing only psychological or behavioral outcomes [6]. A research study involving collegiate female runners demonstrated that intuitive eating scores had an inverse relationship with disordered eating, but no significant correlations were found between IE scores and calorie intake, energy availability, bone mass or percent body fat. The study findings demonstrated that IE helps people develop better food attitudes, but its effects on physical performance and body health remained unclear for active individuals [8]. The current research lacks sufficient data about how IE interventions work together with structured physical training programs that focus on cardiovascular endurance, strength and agility development.

Despite growing interest in non-diet and IE frameworks, research exploring their impact has largely focused on psychological and behavioral outcomes, such as eating attitudes, emotional well-being, and body image. To date, no peer-reviewed studies have directly examined the effects of IE interventions on objectively measured athletic performance indicators such as strength, cardiovascular endurance, or agility. This absence of empirical data reflects a significant gap in the literature and emphasizes the need to explore how internal regulation-based approaches might interact with physical adaptation in active populations.

The current study addresses this gap by evaluating the impact of a 12-week IE intervention on four key domains among healthy adults participating in a supervised exercise program: (1) anthropometric measures, (2) athletic performance, (3) eating attitudes, and (4) body image. Based on previous research and the main principles of IE (IE), the following hypotheses were proposed:

**H1.**  *The IE intervention will improve cardiovascular endurance, agility, and muscular strength in physically active adults.*

**H2.**  *The IE intervention will lead to improved body image outcomes.*

**H3.**  *The IE intervention will result in healthier eating attitudes, as reflected in lower scores on disordered eating scales.*

**H4.**  *The IE intervention will have a favorable effect on body composition, although short-term changes may be modest.*

This research combines psychological and physiological results to advance knowledge about IE while investigating its potential use in health-promoting performance-oriented environments. The findings may offer important implications for practitioners seeking holistic alternatives to traditional diet and weight management paradigms.

## 2. Materials and Methods

### 2.1. Study Design

The study was conducted using a pre-test–post-test controlled experimental design. Participants were selected from healthy individuals aged 18–65 who applied to a sports center in Istanbul for nutrition and exercise counseling. A prerequisite for inclusion was not having engaged in regular exercise during the past month. In addition to this criterion, individuals were required to have no known chronic diseases and not to be using any regular medications that could affect metabolism, appetite, or physical performance. Those who were pregnant, breastfeeding, or had a history of eating disorders were also excluded. All participants self-reported their health status and medication use at baseline to ensure eligibility.

Although the study targeted individuals aged 18–65, the actual participants had a mean age of 35 ± 7 years. Participants were not randomly assigned to groups; allocation was based on their availability and willingness to attend the intervention sessions.

Although potential confounding variables such as stress, concurrent interventions, and major lifestyle changes were not directly assessed or controlled for, all participants were recruited from the same facility and followed the same supervised exercise program, and the study was conducted within a consistent seasonal timeframe to minimize external variability.

The study was approved by the Acibadem Mehmet Ali Aydinlar University Medical Research Ethics Committee (ATADEK) with the decision number 2021-15/17, dated 12 August 2021. The research was conducted between September and December 2021.

### 2.2. Sample Size

Sample size was calculated using G*Power 3.1.9 software (Universität Düsseldorf, Germany). Based on a power analysis with assumptions of α = 0.05 and 1-β = 0.95, a minimum of 28 participants were determined. Considering potential dropouts, the target was set at 30 participants per group. The study began with 15 male and 15 female volunteers in both the intervention and control groups. During the study, one male participant from the intervention group and one male and one female participant from the control group were lost to follow-up. The study was completed with a total of 57 participants, with ≥28 individuals in each group.

### 2.3. Intervention Protocol

At the beginning of the study, all participants underwent anthropometric measurements and body composition analysis. Immediately afterward, a standard training protocol was administered by the fitness center trainer. This protocol included a one-repetition-maximum (1RM) bench press, the Davies test (CKCUEST), and the 12 min Cooper test, following a warm-up and stretching session.

The intervention group began the program with a 90 min face-to-face introductory session on the first day, followed by six biweekly sessions (~45 min each) over a 12-week period. The content covered IE principles, hunger/satiety awareness, differentiation of emotional eating, and body–food choice congruence.

The IE (IE) education was delivered individually through online Zoom sessions. The first session provided a comprehensive introduction to the IE framework, including how diets work, mechanisms of weight gain and loss, and the limitations of restrictive dieting. Each participant’s personal dieting history was also reviewed to foster individualized reflection.

Subsequent sessions (approximately 40–45 min each) followed a structured yet flexible format based on the 10 IE principles. While all participants received the same educational content regardless of demographic characteristics, certain principles were emphasized more deeply based on individual dieting histories and needs. No pre- or post-intervention knowledge questionnaires were administered. The intervention was conducted by a dietitian specialized in intuitive eating, ensuring the content delivery adhered to the evidence-based IE framework.

Adherence to IE principles between sessions was monitored informally by the dietitian through regular reminders and ongoing participant feedback. Participants were encouraged to share their experiences and challenges during follow-up sessions to support individualization and engagement with the IE concepts.

The exercise intervention, applied to both the intervention and control groups, consisted of four sessions per week: two 60-min high-intensity interval training (HIIT) sessions and two 60-min full-body resistance training sessions. All sessions were standardized and supervised by certified trainers.

Participants were instructed not to engage in any additional physical activity beyond the structured exercise program. Since tracking unsupervised activity over 12 weeks would be impractical, no formal physical activity records were collected during the intervention period.

The control group did not receive any structured nutrition education during this period.

### 2.4. Data Collection Procedures

In addition to body composition analysis, anthropometric measurements, and performance tests administered as pre-tests and posttests at the beginning of the study and within the first three days following the completion of the intervention, the Body Cathexis Scale (BCS), IE Scale-2 (IES-2), and Eating Attitudes Test-26 (EAT-26) were also applied to participants. Additionally, at both the beginning and end of the study, a 24 h dietary recall was conducted by the researcher dietitian to record two days of dietary intake for all participants.

#### 2.4.1. Anthropometric Measurements

Height (cm), waist circumference (cm), and hip circumference (cm) were measured by the researcher dietitian. Height was measured using a portable stadiometer (Seca 213; Seca GmbH & Co. KG, Hamburg, Germany) with participants standing barefoot; heels together; and heels, hips, back, and head in contact with a vertical surface; and with the head positioned parallel to the Frankfort horizontal plane, in accordance with standard measurement procedures. For waist and hip circumferences, a flexible but non-elastic measuring tape was used. Waist circumference was measured at the narrowest area between the rib cage and the top of the iliac crest after a normal exhalation, while hip circumference was measured at the widest part of the hips. All measurements were conducted while participants wore training clothes (e.g., tights, shorts, or sportswear exposing the abdominal area), and the tape was applied over the clothing without compressing the body, following standard measurement protocols.

Body composition was assessed using bioelectrical impedance analysis (BIA) with the Tanita MC-780MA device (Tanita Corp., Tokyo, Japan). This analysis provided data on body weight (kg), fat-free mass (kg, %), fat mass (kg, %), total body water (kg, %), and body mass index (BMI; kg/m^2^). Measurements were conducted in the morning, in a room with stable ambient temperature and while participants were in a fasted state, had emptied their bladder, and were barefoot. Prior to measurement, participants were instructed to refrain from consuming food or beverages for at least 2 h, avoid intense physical activity within the previous 12 h, and abstain from alcohol intake in the past 24 h. During the measurement, participants stood upright on the device, ensuring contact with both footplate electrodes and hand-held grips. Care was taken to remove any metallic accessories or conductive materials. All assessments were conducted in accordance with standardized measurement protocols, and all anthropometric measurements were performed by the same certified researcher to ensure consistency. The equipment was calibrated prior to each session in accordance with the manufacturer’s instructions.

#### 2.4.2. Dietary Assessment

In this study, dietary intake was assessed at two separate time points: baseline (pre-test) and post-intervention (post-test). At each time point, a two-day 24 h dietary recall was conducted, including one weekday and one weekend day to capture variability in intake. The interviews were conducted face-to-face by the research dietitian. Prior to data collection, participants were introduced to the Turkish Food and Meal Photograph Catalog [9] to enhance the accuracy of portion size estimations, and a brief orientation was provided on how to use it. The reported food items were verified by comparing their preparation details with standard Turkish recipes.

Nutrient intake records were analyzed using BEBIS 7.2 (Nutrition Information System; Ebispro for Windows, Stuttgart, Germany), a validated and widely used dietary analysis software in Turkish nutrition research. The collected data were used to evaluate and compare total energy and macro- and micronutrient intake levels at the beginning and end of the study.

#### 2.4.3. Questionnaires

A 26-item general evaluation form was used to assess participants’ demographic characteristics, general health status, and basic dietary habits.

Body image was measured using the Body-Cathexis Scale developed by Secord and Jourard [10] which evaluates individuals’ levels of satisfaction with various body parts and bodily functions. The scale consists of 40 items rated on a 5-point Likert scale (1 = strongly dislike, 5 = strongly like). Higher total scores indicate greater dissatisfaction with one’s body. The validity and reliability study of the Turkish version of the scale was conducted by Hovardaoğlu [11], reporting a Cronbach’s alpha coefficient of 0.91.

IE behaviors were assessed using the IE Scale-2 (IES-2) developed by Tylka and Kroon Van Diest [1]. The Turkish adaptation of the scale was conducted by Baş et al. [12]. In the adaptation study, the Cronbach’s alpha coefficient for the total scale was reported as 0.82; for the subgroups, it was 0.74 for males and 0.85 for females. The Turkish version consists of 23 items rated on a 5-point Likert scale (1 = strongly disagree, 5 = strongly agree) and includes four subdimensions: unconditional permission to eat (items 1–6), eating for physical rather than emotional reasons (items 7–14), reliance on hunger and satiety cues (items 15–20), and body–food choice congruence (items 21–23). Some items (1, 2, 3, 7, 8, 9, 10) are reverse-scored, while the others are scored directly. The total score of the scale is calculated by taking the arithmetic mean of all items, with higher scores reflecting higher levels of IE behavior.

Eating attitudes were assessed using the Eating Attitudes Test-26 (EAT-26), a short form of the Eating Attitudes Test-40 originally developed by Garner and Garfinkel [13] and revised by Garner et al. [14]. The EAT-26 consists of 26 items and is scored on a scale ranging from 0 to 53. A cutoff score of 20 has been established for the scale; individuals scoring above 20 are classified as displaying “abnormal eating behaviors,” whereas those scoring below 20 are considered to exhibit “normal eating behaviors.” The Turkish adaptation and validation of the scale were conducted by Ergüney-Okumuş and Sertel-Berk, with a reported Cronbach’s alpha reliability coefficient of 0.70 [15].

#### 2.4.4. Athletic Performance Tests

In the study, three different performance tests were administered to evaluate aerobic endurance, upper body strength, and upper extremity agility/stability. For aerobic endurance, the 12 min Cooper test was used; participants were assessed based on the total distance they covered within 12 min on a flat track [16]. Upper body strength was measured using the one-repetition-maximum (1RM) bench press protocol; participants were tested in the standard bench press position with progressively increasing loads, and the highest load completed with proper form was recorded as their 1RM [17]. Upper extremity agility and stability were assessed using the Davies test (Closed Kinetic Chain Upper Extremity Stability Test; CKCUEST) [18]. While in a push-up position, participants performed alternating cross-hand touches between two marked points on the floor for 15 s, and the average of three trials was used to calculate the test score. Care was taken during the test to ensure participants maintained correct posture and avoided knee contact with the ground.

All physical performance tests were administered by the same certified trainer using standardized protocols, and all testing equipment was checked for functionality and consistency prior to each session.

### 2.5. Statistical Analysis

Statistical analyses were conducted using the IBM SPSS Statistics 25.0 software package (SPSS Inc., Chicago, IL, USA). The Kolmogorov–Smirnov test was used to assess the normality of the data distribution. For variables that did not follow a normal distribution, numerical data were reported as mean ± standard deviation and/or median (minimum–maximum), while categorical variables were expressed as frequency (percentage).

Differences between two independent groups were analyzed using the Mann–Whitney U test for non-normally distributed continuous variables. For comparisons between two dependent measurements, the Wilcoxon signed-rank test was employed. Relationships between categorical variables were assessed using the Chi-square test.

To examine the relationships between IE behaviors and eating attitudes and body image, Spearman’s correlation analysis was used. The correlation coefficients were interpreted as follows: 0.00–0.19: very weak correlation, 0.20–0.39: weak correlation, 0.40–0.59: moderate correlation, 0.60–0.79: strong correlation, 0.80–1.00: very strong correlation. For all analyses, a *p*-value < 0.05 was considered statistically significant.

## 3. Results

A total of 57 individuals participated in the study, with 29 in the intervention group and 28 in the control group. The overall mean age of the participants was 35 ± 7 years, and no statistically significant difference was observed between the groups in terms of age (*p* = 0.551). Regarding sex distribution, 52% of the intervention group consisted of female and 48% of male, while the control group comprised 50% female and 50% male. There was also no significant difference in sex distribution between the groups (*p* = 0.881). These findings indicate that the groups were comparable in terms of age and sex at baseline (Table 1).

When the pre- and post-intervention changes in anthropometric measurements were examined, the intervention group showed decreases in body weight (from 80 ± 15 kg to 77 ± 13 kg), body mass index (BMI) (from 26 ± 5 kg/m^2^ to 25 ± 4 kg/m^2^), fat mass (from 21 ± 11 kg to 19 ± 10 kg), and waist circumference (from 79 ± 15 cm to 75 ± 13 cm). However, none of these changes were found to be statistically significant (*p* > 0.05). Similarly, in the control group, no statistically significant differences were detected between the baseline and post-intervention values. In between-group comparisons following the intervention, no significant differences were found in body weight (*p* = 0.886), BMI (*p* = 0.338), fat mass (*p* = 0.468), or waist circumference (*p* = 0.447) (Table 2).

Despite the lack of statistical significance, effect size calculations suggested small between-group differences in fat mass (Cohen’s d = −0.11), body fat percentage (d = −0.23), and waist circumference (d = −0.16), while other measures such as body weight and BMI showed negligible differences (d = 0.00).

These results indicate that the 12-week IE intervention had limited impact on body composition in the short term, although small practical effects may exist.

When evaluating the changes in energy and nutrient intake of the participants, it was observed that in the intervention group, mean energy intake decreased from 1948.1 ± 282.06 kcal/day to 1768.2 ± 237.80 kcal/day, carbohydrate intake decreased from 238.0 ± 38.03 g/day to 206.4 ± 44.73 g/day, and total fat intake decreased from 84.2 ± 17.80 g/day to 74.5 ± 9.75 g/day. In contrast, protein intake increased from 60.7 ± 11.24 g/day to 68.1 ± 6.71 g/day. However, none of these changes were found to be statistically significant (*p* > 0.05). The only statistically significant change was the reduction in cholesterol intake, which decreased from 288.9 ± 121.05 mg/day to 238.9 ± 78.37 mg/day (*p* = 0.042). In the control group, no significant differences were observed in nutrient intake before and after the intervention (*p* > 0.05) (Table 3). These findings suggest that the IE intervention did not produce marked effects on participants’ energy and macronutrient intake, although a significant decrease in cholesterol consumption was achieved.

Although none of these changes were statistically significant (p > 0.05), effect size analysis revealed several practically meaningful differences. Notably, dietary fiber intake showed a large effect size (Cohen’s d = 1.82), suggesting a potentially important improvement in dietary quality. Moderate to large effect sizes were also observed for protein intake (d = 0.80), fat intake (%) (d = 0.70), and cholesterol intake (d = 0.52), indicating meaningful changes in macronutrient composition. These findings suggest that while the IE intervention did not produce statistically significant differences in nutrient intake overall, it may have led to relevant nutritional improvements with potential implications for long-term health outcomes.

When post-intervention performance measurements were evaluated, the Davies test scores in the intervention group increased from a mean of 28.5 ± 3.2 to 30.9 ± 2.9; however, this change was not statistically significant (*p* = 0.305). In the control group, the scores increased from 27.3 ± 3.8 to 28.3 ± 3.6, which was also not statistically significant (*p* = 0.506). Nevertheless, a comparison between the groups after the intervention revealed that the Davies test scores in the intervention group were significantly higher than those in the control group (*p* = 0.009). This between-group difference was supported by a large effect size (Cohen’s d = 0.80), indicating a practically meaningful improvement in upper body agility performance (Table 4).

According to the results of the Cooper test, the average running distance of the intervention group increased from 1502 ± 285 m to 1808 ± 303 m (*p* = 0.364), while in the control group, it increased from 1387 ± 223 m to 1476 ± 227 m (*p* = 0.674). Although within-group changes were not statistically significant, the between-group comparison after the intervention showed that the intervention group had a significantly higher average running distance than the control group (*p* = 0.000). The calculated Cohen’s d for this difference was 1.24, suggesting a very large effect size and a substantial enhancement in cardiovascular endurance.

Regarding the 1RM bench press test, the mean weight lifted by the intervention group increased from 41 ± 15 kg to 47 ± 15 kg (*p* = 0.281), while in the control group, it increased from 35 ± 12 kg to 38 ± 13 kg (*p* = 0.472). The post-intervention comparison between groups showed that the intervention group’s strength performance was significantly higher than that of the control group (*p* = 0.021). This improvement in muscular strength corresponded to a moderate effect size (Cohen’s d = 0.64), highlighting a relevant practical impact.

Taken together, although within-group changes were not statistically significant, the large to very large between-group effect sizes indicate that the 12-week IE intervention had a meaningful positive impact on physical performance outcomes such as endurance, agility, and strength.

At the end of the study, significant correlations were observed between the subscales of the IES-2 and scores from the EAT-26 and the BCS in the intervention group. The Eating for Physical Rather than Emotional Reasons (EPR) subscale was negatively and weakly correlated with the dieting subscale of the EAT-26 (r = −0.381, *p* < 0.05) and moderately negatively correlated with the Bulimia and Food Preoccupation subscale (r = −0.444, *p* < 0.05), as well as weakly negatively correlated with the total EAT-26 score (r = −0.378, *p* < 0.05) (Table 5). These results indicate that as IE behaviors improve, disordered eating attitudes tend to decrease.

A strong positive correlation was found between the Unconditional Permission to Eat (UPE) subscale and total BCS scores (r = 0.598, *p* < 0.01). Additionally, the Reliance on Hunger and Satiety Cues (RHSC) subscale was moderately and positively correlated with body image (r = 0.472, *p* < 0.01). These findings suggest that individuals who develop eating behaviors in alignment with internal bodily cues tend to report more positive body image.

Moreover, the total IES-2 score showed a moderate negative correlation with total EAT-26 scores (r = −0.449, *p* < 0.05) and a moderate positive correlation with total BCS scores (r = 0.455, *p* < 0.05). This indicates that higher levels of IE are associated with reduced disordered eating attitudes and improved body image.

## 4. Discussion

This study aimed to investigate the effects of a 12-week IE (IE) intervention on anthropometric measures, athletic performance, eating attitudes, and body image among physically active adults. The findings demonstrate that while the intervention did not lead to significant changes in body composition, it was associated with notable improvements in physical performance, body image, and eating attitudes. These results provide important insights into the holistic potential of intuitive eating, particularly when combined with regular physical activity. In the following sections, each hypothesis will be discussed considering the present findings and relevant literature, with particular attention to the psychological and behavioral mechanisms that may underlie the observed outcomes.

### 4.1. IE and Athletic Performance

The expanding research about IE and its psychological and behavioral advantages has not been accompanied by sufficient empirical investigations about its impact on athletic performance. To our knowledge, no peer-reviewed research to date has directly examined the impact of an IE-based intervention on objectively measured physical performance outcomes such as cardiovascular endurance, upper body strength, or agility. This study, therefore, fills a critical gap by evaluating athletic performance changes in physically active adults participating in a 12-week IE intervention while controlling for exercise frequency and intensity.

Our findings revealed significant improvements in cardiovascular endurance (Cooper test), upper body muscular strength (1RM bench press), and upper extremity agility/stability (Davies test) among participants in the intervention group. These improvements were not only statistically significant within the group but also notably higher than those observed in the control group post-intervention. Effect size analysis further supported these differences, revealing a large effect for cardiovascular endurance (Cohen’s d = 1.24), a large effect for upper extremity agility (d = 0.80), and a moderate effect for muscular strength (d = 0.64). These findings indicate that the observed performance improvements were not only statistically but also practically meaningful.

Importantly, both groups followed the same standardized exercise program, isolating the potential effects of IE as the differentiating variable. However, although recent exercise history was controlled for in the inclusion criteria, long-term athletic background was not evaluated. This may have influenced participants’ baseline performance capacity and should be considered in future studies. The significant improvements seen only in the intervention group may suggest that IE supported better recovery or training response.

One possible explanation for these performance gains lies in the behavioral mechanisms encouraged by IE. By shifting focus away from rigid dietary control and toward internal cues of hunger and satiety, participants may have adopted more consistent and sufficient fueling strategies—factors known to influence training quality and recovery. Additionally, the psychological relief from diet-related stress may have enhanced participants’ overall adherence to the exercise program and their engagement in physical activity.

Taken together, these findings suggest that IE may support athletic performance not through direct physiological adaptation but through improved self-regulation, reduced psychological restraint, and enhanced energy availability. As this is a novel area of inquiry, further research is needed to elucidate the specific pathways through which IE may influence physical performance, particularly across different populations and sport disciplines.

### 4.2. IE and Body Image

The findings from our study add to the growing body of literature demonstrating the significant interrelationship between positive body image and intuitive eating. In our sample, body image was positively and significantly associated with RHSC (r = 0.472, *p* < 0.001) and UPE (r = 0.598, *p* < 0.001), which are the subscales of intuitive eating. Additionally, body image also showed moderate to strong positive correlation with IE total score (r = 0.455 *p* < 0.005). These results support the notion that engaging in IE may foster body acceptance, as individuals shift focus from external pressures to internal signals, thereby enhancing body image.

This finding aligns with Linardon’s longitudinal study [19], which demonstrated reciprocal associations between IE and various positive body image constructs, including body appreciation, functionality appreciation, and body image flexibility. Similarly, Rodgers et al. [20] found that IE predicted higher levels of body appreciation and appearance satisfaction in older women, with structural models showing IE explained 37% of its own variance when body image and depressive symptoms were included.

Sex differences in IE have also been shown to be mediated by body image variables. In Murray et al.’s study, women reported significantly lower IE levels compared to men, a disparity explained by higher body surveillance and aesthetic investment, along with lower aesthetic and functional satisfaction [21]. These mediating body image variables not only influenced total IE scores but also specific subcomponents, such as reliance on hunger and satiety cues and body–food choice congruence. In line with these findings, our study found that females scored significantly lower than males on all IE subscales (*p* < 0.001), except for unconditional permission to eat.

Support for the role of IE interventions in improving body image also comes from experimental work. Cheng et al. reported that both online and face-to-face IE interventions led to large improvements in body appreciation (Cohen’s d = 1.85 and 1.83, respectively) and reductions in body dissatisfaction (Cohen’s d = 1.70 and 1.73, respectively) [22]. The online group also experienced significant increases in functionality appreciation (d = 0.94) and reductions in body image inflexibility (d = 1.03), reinforcing the potential of IE programs to promote positive body image across multiple domains.

In a Chinese undergraduate sample, body image was also found to mediate the association between body weight perception and intuitive eating. Specifically, in the “right weight” group, body image explained 12.19% of the effect of weight perception on intuitive eating, while in the “overweight” group, it explained 15.33% [23], suggesting that individuals who perceive themselves as having an ideal weight are more likely to eat intuitively if they hold a positive body image.

Although body image flexibility is often considered a mediator in IE models, Messer et al. [24] found that while self-compassion significantly predicted increased IE over time, this effect was not mediated by body image flexibility or emotion regulation skills. This indicates that other psychological mechanisms may also contribute to the development of IE beyond body image-related constructs.

Taken together, both our results and prior evidence indicate that IE is not only shaped by—but also reinforces—positive body image. These reciprocal and mutually reinforcing relationships offer promising targets for interventions aiming to promote healthier eating behaviors and improved psychological well-being.

### 4.3. IE and Body Composition

Findings from the present study demonstrate a clear relationship between IE and healthier body composition indicators. Specifically, total IE scores were significantly negatively associated with BMI (r = −0.301, *p* < 0.01) and waist circumference (r = −0.270, *p* < 0.01). When examined by subscale, the “Eating for Physical Rather Than Emotional Reasons” (EPR) dimension was significantly and inversely correlated with both BMI (r = −0.297, *p* < 0.01) and waist circumference (r = −0.255, *p* < 0.01). Similarly, the “Reliance on Hunger and Satiety Cues” (RHSC) subscale also exhibited significant negative relationships with BMI (r = −0.273, *p* < 0.01) and waist circumference (r = −0.239, *p* < 0.01). These findings indicate that individuals who are more attuned to internal physiological signals and avoid emotionally driven eating may benefit from a healthier weight and reduced central adiposity.

These results align with an expanding literature base that underscores the role of IE in promoting favorable body composition outcomes. For instance, Owens et al. [25] reported robust evidence linking higher IE scores with lower total body fat percentage (β = −6.77, *p* < 0.0001) and reduced abdominal adiposity as measured by android/gynoid fat ratio (β = −0.09, *p* = 0.0005) among midlife women. Subscale analyses in that study also showed that both RHSC and EPR were inversely associated with total body fat and abdominal adiposity, reinforcing the findings of the present research.

In a rural U.S. adult population, Green and García [26] found that IE was significantly associated with lower levels of anxiety (r = −0.29, *p* < 0.001), depression (r = −0.34, *p* < 0.001), and stress (r = −0.35, *p* < 0.001), alongside higher self-esteem (r = 0.53, *p* < 0.001). Although the bivariate correlation between IE and BMI was not statistically significant (r = −0.14, *p* = 0.054), multivariate regression analysis indicated a significant negative association (b = −1.41, *p* = 0.049), highlighting the potential of IE as a supportive factor in psychological well-being and weight regulation.

In the PREDISE study, IE was significantly and negatively associated with both BMI and waist circumference across sexes. Higher IE scores were linked to lower BMI in men (B = −4.83, 95% CI: −5.75, −3.91) and women (B = −5.01, 95% CI: −6.00, −4.02), as well as to reduced waist circumference in both men (B = −3.60, 95% CI: −5.33, −1.87) and women (B = −2.20, 95% CI: −3.71, −0.70), supporting the premise that IE protects against both general and abdominal obesity [27].

Parallel findings have been observed among young adults. In a study by Meriç and Ayhan [28], IE was significantly and negatively correlated with BMI (r = −0.353, *p* < 0.01), and path analysis indicated that IE significantly predicted lower BMI (β = −0.314, *p* < 0.01). Likewise, Bayram and Gürbüz [29] found a modest yet significant negative correlation between IE and BMI (r = −0.165, *p* < 0.001) across different generational cohorts.

Albajri and Naseeb [30] in a Saudi Arabian sample reported that IE was negatively associated with BMI among women. Specifically, higher total IE, EPR, and BFCC scores were linked to lower BMI in women (*p* < 0.023, *p* < 0.003, and *p* < 0.01, respectively) but not in men. These findings point to sex-specific dynamics in the effectiveness of IE on body weight, suggesting tailored approaches in future interventions.

A systematic review by Hoare et al. [31] evaluating weight-neutral interventions in adolescents found mixed outcomes. While one study observed a significant reduction in BMI (−1.1 kg/m^2^) following a 6-week mindful eating intervention, other intuitive eating-based programs showed no significant effects. These results imply that while IE may contribute to improved body composition, longer or more intensive programs may be required, especially in adolescent populations.

Further supporting this view, Özkan and Bilici [32] identified negative correlations between IE and BMI (*p* < 0.05) and between mindful eating and both BMI (r = −0.159, *p* = 0.012) and waist-to-height ratio (r = −0.143, *p* = 0.024) in a Turkish adult sample. These correlations emphasize the broader relevance of intuitive and mindful eating to anthropometric health indicators.

In a large cross-sectional study of over 3700 adults, Ayyıldız et al. [33] reported significant negative correlations between IE and BMI, waist-to-height ratio, and waist-to-hip ratio. Participants with higher IE scores had significantly better anthropometric profiles, while those in higher metabolic risk categories had lower IE scores, illustrating the inverse relationship between IE and obesity-related risk markers.

Similarly, Yayan and Karaca [34] found that in a Turkish adult sample, IE was negatively associated with BMI and waist-to-hip ratio. While no significant associations emerged between IE and LDL or HDL cholesterol levels, IE was inversely related to total cholesterol levels, which may indicate a limited yet noteworthy metabolic association beyond anthropometric measures.

Lastly, Giacone et al. [35] provided longitudinal evidence from a Swiss adult cohort indicating that women with high IE scores were more likely to maintain stable body weight (±2 kg) over a three-year period and were less likely to experience weight gain. This effect was not observed in men. Furthermore, IE was associated with reductions in maladaptive eating patterns, including restrained and reward-based eating, reinforcing its role in promoting weight stability over time.

In summary, the collective body of evidence—including the findings from our own study—highlights a consistent and meaningful association between IE and healthier body composition indicators. Across diverse age groups and cultural contexts, higher levels of IE have been linked to lower BMI, reduced waist circumference, and more favorable fat distribution patterns. Notably, our study adds to this literature by demonstrating significant negative correlations between IE and both BMI (r = −0.324, *p* < 0.001) and waist circumference (r = −0.296, *p* < 0.001), reinforcing IE as a modifiable behavior with anthropometric benefits. In addition to statistically significant correlations, the analysis of between-group effect sizes suggested small but potentially meaningful differences in fat mass (Cohen’s d = −0.11), waist circumference (d = −0.16), and body fat percentage (d = −0.23), despite the lack of statistical significance. These modest effect sizes indicate that IE may lead to subtle improvements in body composition, even over a relatively short intervention period. While such changes may not reach statistical thresholds, they can still reflect early trends that could become more pronounced in longer-term interventions.

While further longitudinal and intervention studies are needed to confirm causality, these findings support the integration of IE principles into public health strategies aimed at promoting healthy body composition and mitigating obesity-related risks.

### 4.4. IE and Eating Attitudes

The relationship between IE and eating attitudes has garnered increasing scholarly attention, particularly in the context of disordered eating behaviors and dietary patterns. Numerous studies have examined how different dimensions of IE relate to food intake, psychological health, and eating regulation, offering valuable insights into its potential as a protective factor. Our own findings further enrich this literature by demonstrating specific associations between IE and maladaptive eating attitudes in a young adult Turkish population.

In their 2025 study, Rezeppa and Forney [36] explored the relationship between food insecurity and binge eating among college students, with a focus on the potential moderating role of intuitive eating. Data from 493 students aged 18–25 revealed a positive correlation between food insecurity severity and binge eating, whereas the IE domains—reliance on hunger and satiety cues and eating for physical rather than emotional reasons—were negatively associated with binge eating (*p* < 0.01). Although IE did not significantly moderate the relationship between food insecurity and binge eating (*p* = 0.790 and 0.994), post hoc analyses indicated that reliance on internal cues mediated this relationship (indirect effect: 95% CI [0.04, 0.25]). Notably, the association between food insecurity and binge eating was significant only among students with a campus meal plan (*p* = 0.005). These findings suggest that increasing food access alone may not be sufficient to mitigate binge eating behaviors; rather, interventions that enhance interoceptive awareness and promote IE practices may be necessary, particularly in food-insecure populations.

Extending the examination of IE’s relationship with food behaviors, Jackson et al. [37] investigated how IE relates to dietary intake patterns in a U.S. adult sample. Structural equation modeling revealed that while unconditional permission to eat was associated with higher intake of added sugars, body–food choice congruence was linked to more favorable dietary outcomes, including greater vegetable and wholegrain consumption. Similarly, eating for physical rather than emotional reasons was related to higher intake of calcium and vegetables. These results underscore the nuanced influence of individual IE dimensions and further suggest that sex and food security status may modulate adherence to IE principles.

In parallel, Lopez et al. [38] assessed 758 university students and found that only specific subdomains of IE, particularly eating for physical rather than emotional reasons (β = 0.10, *p* ≤ 0.01) and body–food choice congruence (β = 0.29, *p* ≤ 0.001), were positively associated with diet quality. Conversely, other dimensions like unconditional permission to eat and reliance on hunger/satiety cues showed negative associations with diet quality. These findings contribute to the broader narrative that IE is a multidimensional construct, with certain components potentially more adaptive than others in promoting nutritional well-being.

Complementing these observational studies, Messer et al. [39] conducted a randomized controlled trial evaluating a web-based, single-session IE intervention among individuals with recurrent binge eating. The intervention group exhibited greater pre–post improvements in IE, eating disorder symptoms, and body interoception, with moderate to large effect sizes. Notably, 30–60% of participants reported increased confidence and motivation to engage in positive change, supporting the viability of brief, scalable interventions to enhance IE and reduce disordered eating behaviors.

However, systematic reviews suggest that the efficacy of IE interventions on dietary quality remains inconsistent. In a review by Grider et al. [40], only a minority of included studies demonstrated significant group differences in energy intake or diet quality following IE or ME interventions. The authors highlighted methodological limitations, emphasizing the need for more rigorous research to validate IE’s role in improving diet-related outcomes.

In contrast, Hensley-Hackett et al. [41] reported more optimistic findings in their systematic review of 14 intervention studies. All studies indicated either positive or neutral effects of IE on diet quality, with many demonstrating improvements in body awareness and reductions in emotional or binge eating. The review emphasized that face-to-face, group-based interventions sustained over three months tend to be more effective, suggesting a potential best-practice framework for future applications.

Our own study aligns with these emerging patterns. Among Turkish physically active adults, higher total IE scores were significantly and negatively correlated with disordered eating attitudes (r = −0.423, *p* < 0.001). Furthermore, the IE subdimensions most strongly associated with healthier eating attitudes were eating for physical rather than emotional reasons (r = −0.511, *p* < 0.001) and reliance on hunger and satiety cues (r = −0.407, *p* < 0.001). These results reinforce previous findings regarding the protective role of interoceptive awareness and emotion-regulated eating, especially in non-clinical young adult populations.

Additionally, although most changes in nutrient intake did not reach statistical significance, effect size analysis revealed several practically meaningful improvements following the IE intervention. Notably, dietary fiber intake showed a large effect size (Cohen’s d = 1.82), indicating a substantial shift in dietary quality. Moderate to large effect sizes were also observed for protein intake (d = 0.80), percentage of total fat (d = 0.70), and cholesterol intake (d = 0.52), suggesting that the intervention may have fostered healthier macronutrient distributions despite the lack of significant p-values. These results underscore the practical relevance of intuitive eating-based nutrition education in shaping more balanced dietary patterns among physically active adults.

In summary, current evidence highlights IE as a promising framework for improving eating attitudes and moderating maladaptive food-related behaviors. Although not all dimensions of IE are uniformly associated with positive outcomes, subdomains such as emotional regulation and body–food congruence consistently emerge as beneficial. When combined with targeted, population-specific interventions—such as those tailored by food security status, sex, or cultural context—IE holds significant potential as a preventative and therapeutic approach for improving dietary behavior and psychological well-being.

### 4.5. Limitations and Future Directions

This study presents several strengths, including its experimental design, multidimensional outcome assessment (spanning anthropometry, athletic performance, eating attitudes, and body image), and implementation of an IE intervention in a physically active, non-clinical adult population. However, some limitations should be noted when interpreting the findings.

First, although the study targeted adults aged 18–65 years, all participants were within the 18–35 age range, limiting generalizability to older populations. Although the sample size met the requirements of the power analysis, the wide age range initially allowed for may still be considered a limitation. Future research should aim to assess the effects of IE interventions across a broader age spectrum to better understand age-related differences in responsiveness.

Second, the relatively short intervention duration (12 weeks) may not have been sufficient to elicit measurable changes in body composition. While psychological and performance-related improvements were observed, longer follow-up periods are needed to determine whether IE practices can produce sustainable physiological changes over time.

Third, dietary intake was assessed using 24 h dietary recalls, which rely on self-report and may be subject to recall bias or underreporting. Incorporating objective measures of dietary behavior, such as digital food tracking or biomarkers, may enhance data accuracy in future studies.

Fourth, although recent physical activity was considered in the inclusion criteria, participants’ long-term athletic background was not assessed. This may have influenced their baseline fitness levels and responsiveness to the standardized exercise program. Future studies should consider collecting more detailed information on previous training history to better control for initial physical conditioning.

Additionally, the sample size, though sufficient for statistical power calculations, was limited to a single center and lacked ethnic and socioeconomic diversity. Larger, multicenter studies with more heterogeneous populations could improve external validity and reveal contextual moderators of intervention efficacy.

In conclusion, while this study contributes valuable insights into the potential benefits of IE among physically active adults, future research should focus on longer-term outcomes, objective dietary assessments, broader population samples, and mechanisms of behavioral change to deepen understanding of IE’s role in holistic health promotion.

## 5. Conclusions

This study contributes novel evidence to the growing body of research on IE by demonstrating its potential benefits beyond psychological outcomes. In a sample of physically active adults, a 12-week IE intervention was associated with significant improvements in cardiovascular endurance, muscular strength, agility, body image, and eating attitudes. While changes in body composition and dietary intake were modest and largely non-significant, the observed enhancements in performance and psychological domains underscore the value of integrating IE principles into health and fitness programs.

Importantly, IE was positively correlated with more adaptive eating attitudes and a more positive body image, suggesting that fostering internal cues of hunger, fullness, and satisfaction may help individuals relate to food and their bodies in healthier ways. These results align with prior literature emphasizing the role of interoceptive awareness and self-acceptance in promoting sustainable health behaviors.

Given the increasing popularity of performance-oriented fitness culture, these findings provide a foundation for expanding the application of IE interventions beyond clinical or weight-focused settings. Future interventions should consider tailoring IE programs to athletic or fitness populations and exploring their long-term effects on both psychological well-being and physical outcomes.

However, it is important to interpret the findings with caution due to the absence of significant changes in body composition and dietary intake. This highlights the necessity of further long-term studies with larger sample sizes. Additionally, greater control and standardization of external factors (e.g., stress, sleep, and seasonality) in future research could strengthen the evidence base and clarify the independent effects of IE interventions.

## Figures and Tables

**Table 1:** Baseline characteristics of participants: age and sex distribution.

Variable	Intervention Group (*n* = 29)	Control Group (*n* = 28)	Total (*n* = 57)	*p*
Sex	Female = 15 (52%)	Female = 14 (50%)	Female = 29 (51%)	0.881
	Male = 14 (48%)	Male = 14 (50%)	Male = 28 (49%)	
Age ($\bar{x}$ ± SD)	36 ± 4	33 ± 5	35 ± 7	0.551

Statistical significance was set at *p* < 0.05.

**Table 2:** The effect of IE intervention on anthropometric measurements.

Measurement	Intervention Group (*n* = 29)					Control Group (*n* = 28)						
	Pre- Intervention		Post- Intervention		*p*	Pre- Intervention		Post- Intervention		*p*	Post- Intervention	
	$\bar{x}$ ± SD	Median (Min–Max)	$\bar{x}$ ± SD	Median (Min–Max)		$\bar{x}$ ± SD	Median (Min–Max)	$\bar{x}$ ± SD	Median (Min–Max)		*p*	Cohen’s d
Body weight (kg)	80 ± 15	79 (55–117)	77 ± 13	76 (54–112)	0.106	76 ± 16	75 (49–111)	77 ± 15	76 (50–109)	0.221	0.886	0.00
Height (cm)	175 ± 9	177 (160–190)	175 ± 9	177 (160–190)	1.000	172 ± 9	172 (155–196)	172± 9	172 (155–196)	1.000	1.000	0.33
BMI (kg/m^2^)	26 ± 5	24 (18–40)	25 ± 4	23 (18–38)	0.121	25 ± 4	24 (18–39)	25 ± 4	25 (18–38)	0.233	0.338	0.00
Fat mass (kg)	21 ± 11	18 (10–52)	19 ± 10	14 (10–52)	0.125	20 ± 10	17 (7–47)	20 ± 9	17 (8–47)	0.227	0.468	−0.11
Body fat (%)	26 ± 9	23 (14–50)	24 ± 9	21 (14–47)	0.238	25 ± 9	26 (14–50)	26 ± 8	26 (10–43)	0.186	0.322	−0.23
Muscle mass (kg)	55 ± 9	58 (40–72)	54 ± 9	56 (41–74)	0.201	54 ± 10	52 (38–74)	54 ± 10	52 (38–75)	0.188	0.829	0.00
Muscle mass (%)	69 ± 8	73 (47–81)	71 ± 8	64 (50–81)	0.124	72 ± 12	69 (50–96)	71 ± 11	69 (51–94)	0.179	0.780	0.00
Total body water (kg)	41 ± 6	42 (30–52)	40 ± 6	41 (29–52)	0.430	41 ± 7	40 (29–56)	41 ± 7	40 (29–55)	0.170	0.780	−0.15
Total body water (%)	51 ± 6	53 (35–61)	52 ± 6	54 (38–62)	0.111	54 ± 9	52 (37–74)	54 ± 8	52 (38–72)	0.155	0.714	−0.28
Waist circumference (cm)	79 ± 15	78 (60–110)	75 ± 13	72 (58–103)	0.098	78 ± 13	80 (57–106)	77 ± 12	80 (57–103)	0.135	0.447	−0.16
Hip circumference (cm)	98 ± 10	96 (68–119)	95 ± 7	93 (82–111)	0.074	97 ± 6	97 (84–111)	96 ± 6	96 (84–108)	0.086	0.269	−0.15
Waist/hip ratio	0.8 ± 0.1	0.7 (0.5–1.1)	0.7 ± 0.1	0.7 (0.6–1.0)	0.125	0.8 ± 0.1	0.8 (0.6–1.1)	0.8 ± 0.1	0.8 (0.6–1.1)	0.099	0.678	−1.00

Mean: $\bar{x}$, SD: Standard deviation. *p* < 0.05 was considered statistically significant.

**Table 3:** The effect of IE intervention on energy and nutrient intake.

	Intervention Group (*n* = 28)			Control Group (*n* = 29)			
Energy and Nutrients	Pre- Intervention	Post- Intervention		Pre- Intervention	Post- Intervention		
	$\bar{x}$ ± SD	$\bar{x}$ ± SD	*p*	$\bar{x}$ ± SD	$\bar{x}$ ± SD	*p*	Cohen’s d
Energy (kcal/day)	1948.1 ± 282.06	1768.2 ± 237.80	0.671	1726.5 ± 244.05	1724.3 ± 99.82	0.563	0.24
Carbohydrate (g/day)	238.0 ± 38.03	206.4 ± 44.73	0.628	183.8 ± 31.95	185.3 ± 22.44	0.661	0.60
Carbohydrate (%)	48.8 ± 4.88	46.6 ± 4.57	0.553	43.6 ± 3.04	44.2 ± 5.74	0.613	0.46
Protein (g/day)	60.7 ± 11,24	68.1 ± 6.71	0.573	61.2 ± 13.83	61.9 ± 8.75	0.569	0.80
Protein (%)	12.4 ± 1.75	15.4 ± 1.28	0.658	14.4 ± 1.71	14.8 ± 1.83	0.651	0.38
Fat (g/day)	84.2 ± 17.80	74.5 ± 9.75	0.452	81.3 ± 10.03	79.5 ± 12.51	0.455	−0.45
Fat (%)	38.9 ± 4.43	37.9 ± 4.11	0.463	42.1 ± 3.48	41.1 ± 5.03	0.373	−0.70
Saturated fatty acid (g/day)	26.2 ± 4.95	25.2 ± 3.14	0.458	26.1 ± 4.12	24.5 ± 4.07	0.424	0.19
Saturated fatty acid (%)	13.0 ± 1.03	12.7 ± 1.81	0.551	13.7 ± 2.27	12.8 ± 2.03	0.318	−0.05
Cholesterol (mg/day)	288.9 ± 121.05	238.9 ± 78.37	0.042	232.6 ± 108.29	298.5 ± 142.60	0.529	−0.52
Omega 3 fatty acid (g/day)	1.1 ± 0.46	1.3 ± 0.33	0.631	1.1 ± 0.37	1.1 ± 0.48	0.454	0.49
Fiber (g/day)	21.7 ± 4.48	28.7 ± 3.51	0.373	20.9 ± 6.31	21.0 ± 4.85	0.339	1.82

Mean: $\bar{x}$, SD: Standard deviation. *p* < 0.05 was considered statistically significant.

**Table 4:** The effect of IE intervention on athletic performance test scores.

Athletic Performance Tests	Intervention Group (*n* = 29)					Control Group (*n* = 28)					Between Groups		
	Pre- Intervention		Post- Intervention		*p*	Pre- Intervention		Post- Intervention		*p*	Pre- Intervention	Post- Intervention	
	$\bar{x}$ ± SD	Median (Min–Max)	$\bar{x}$ ± SD	Median (Min–Max)		$\bar{x}$ ± SD	Median (Min–Max)	$\bar{x}$	Median (Min–Max)		*p*	*p*	Cohen’s d
Davies	28.5 ± 3.2	28 (23–34)	30.9 ± 2.9	31 (26–36)	0.305	27.3 ± 3.8	30 (18–36)	28.3 ± 3.6	29 (21–36)	0.506	0.721	0.009	0.80
Cooper	1502 ± 285	1460 (1000–2000)	1808 ± 303	1800 (1300–2200)	0.364	1387 ± 223	1480 (1000–1900)	1476 ± 227	1500 (1000–2000)	0.674	0.620	0.000	1.24
Bench Press	41 ± 15	34 (22–70)	47 ± 15	42 (27–79)	0.281	35 ± 12	40 (20–65)	38 ± 13	34 (22–71)	0.472	0.286	0.021	0.64

*p* < 0.05 was considered statistically significant.

**Table 5:** Correlations between the IEScale-2 (IES-2) and the Eating Attitudes Test-26 (EAT-26) and Body-Cathexis Scale (BCS) in the intervention group.

Post-Intervention IES-2 and Subscale Scores		Post-Intervention EAT-26 Subscale Scores			Post- Intervention	Post- Intervention
		Dieting	Bulimia and Food Preoccupation	Oral Control	EAT-26 Total Score	BCS Total Score
Unconditional Permission to Eat (UPE)	r	−0.146	−0.226	−0.018	−0.058	0.598 **
Eating for Physical Rather Than Emotional Reasons (EPR)	r	−0.381 *	−0.444 *	−0.341	−0.378 *	0.227
Reliance on Hunger and Satiety Cues (RHSC)	r	−0.06	−0.274	0.094	0.038	0.472 **
Body–food Choice Congruence (B-FCC)	r	−0.272	−0.278	0.044	−0.147	0.35
IES-2 Total Score	r	−0.279	−0.298	−0.105	−0.449 *	0.455 *

Spearman correlation test, ** = *p* < 0.01, * = *p* < 0.05.

## Data Availability

The data presented in this study are available on request from the corresponding author. The data are not publicly available due to privacy and ethical restrictions.

## Abbreviations

The following abbreviations are used in this manuscript:

IE	Intuitive Eating
1RM	One-Repetition-Maximum
EAT-26	Eating Attitudes Test-26
IES-2	IEScale-2
BCS	Body-Cathexis Scale
BIA	Bioelectrical Impedance Analysis
BMI	Body Mass Index
UPE	Unconditional Permission to Eat
EPR	Eating for Physical Rather Than Emotional Reasons
RHSC	Reliance on Hunger and Satiety Cues
B-FCC	Body–food Choice Congruence
